# Caregiver supportive policies to improve child outcomes in the wake of the HIV/AIDS epidemic: an analysis of the gap between what is needed and what is available in 25 high prevalence countries

**DOI:** 10.1080/09540121.2016.1176685

**Published:** 2016-07-08

**Authors:** Rachel Kidman, Jody Heymann

**Affiliations:** ^a^ Program in Public Health and Department of Family, Population & Preventative Medicine, Stony Brook University, Stony Brook, NY, USA; ^b^ Fielding School of Public Health, University of California Los Angeles, Los Angeles, CA, USA

**Keywords:** HIV-affected children, caregiving, social policy, HIV/AIDS

## Abstract

In the wake of the HIV/AIDS epidemic, caregivers are struggling to support HIV-affected children. For reasons of equity and efficiency, their needs can be best met through strong social protections and policies. This paper presents a conceptual framework to help address the needs of HIV-affected caregivers and to prioritize policies. We describe the needs that are common across diverse caregiving populations (e.g., economic security); the needs which are intensified (e.g., leave to care for sick children) or unique to providing care to HIV-affected children (e.g., ARV treatment). The paper then explores the types of social policies that would facilitate families meeting these needs. We outline a basic package of policies that would support HIV-affected families, and would meet goals agreed to by national governments. We examine the availability of these policies in 25 highly affected countries in sub-Saharan Africa. The majority of countries guarantee short-term income protection during illness, free primary school, and educational inclusion of children with special needs. However, there are significant gaps in areas critical to family economic security and healthy child development. Fewer than half of the countries we analyzed guarantee a minimum wage that will enable families to escape poverty; only six have eliminated tuition fees for secondary school; and only three offer paid leave to care for sick children. Filling these policy gaps, as well as making mental health and social services more widely available, is essential to support caregiving by families for HIV-affected children. As part of the HIV agenda, the global community can help national governments advance towards their policy targets. This would provide meaningful protection for families affected by HIV, as well as for millions of other vulnerable families and children across the region.

Children have been deeply affected by the HIV epidemic. Globally, 2.6 million children are HIV-infected, 13.3 million have been orphaned due to AIDS, and a third of children reside with an HIV-infected adult in the hardest hit countries (Short & Goldberg, 2015; UNICEF, 2015). Families are the primary source of support for HIV-affected children, yet they are too often overwhelmed and under-resourced (Kidman & Heymann, 2009; Kidman & Thurman, 2014; Miller, Gruskin, Subramanian, Rajaraman, & Heymann, 2006). A growing body of literature attests to the economic, psychological, and social strain of caring for HIV-affected children, and to the downstream impact this has on child outcomes. Supporting caregivers and strengthening the family environment in which these children are raised is therefore fundamental to improving child welfare (Richter, 2010; Richter et al., 2009).

Over the past decade, both the global community and individual countries have made progress towards addressing the needs of HIV-affected children and families. Support has most often been delivered through targeted programs, but unfortunately these have not fully been able to meet the needs of this population. National policies and protections offer another way to reach HIV-affected families. Both the United Nations and its member countries have already committed to a broader platform of social protection to address poverty and inequality. This movement has its foundation in the Universal Declaration of Human Rights (UDHR), and encompasses the Convention on the Rights of the Child (CRC), the Convention on the Rights of Persons with Disabilities (CRPD), and the Convention on the Elimination of All Forms of Discrimination against Women (CEDAW) (UN General Assembly, 1948, 1979, 1989). The principles articulated in these conventions – such as the right to health, to education, and to protection from discrimination – are foundational to supporting caregiving.

Recently, a dialog has begun about whether meeting the broader rights enshrined in these international conventions and supported by countries around the world should also be a priority for the HIV sector. HIV-affected caregivers have many of the same needs as other vulnerable caregivers, and would benefit from the same platform of rights and protections. The above conventions are widely endorsed, but rights are realized only through national action; they are operationalized and taken to scale through the adoption of laws and policies. Many high prevalence countries are still working towards these nationally shared targets for various reasons (e.g., financial resources and technical capacity). By embracing social protections as part of their larger agenda, the HIV sector could play an important role in helping governments build on their commitments and support HIV-objectives (Miller & Samson, 2012; The Working Group on Social Protection for the Inter-Agency Task Team on Children and HIV and AIDS, 2008; UNAIDS, 2014; UNICEF, 2012; Yates, Chandan, & Lim Ah Ken, 2010).

This paper brings together new evidence to guide efforts. We begin by exploring how the needs of HIV-affected caregivers intersect with those of other vulnerable caregiving populations and inform our policy response. We then examine the availability of key policies in the 25 most highly endemic countries, highlighting areas of meaningful protection as well as opportunities for greater progress.

## Conceptual framework to guide policy development for HIV-affected caregivers

In this section, we summarize some of the key social, economic, and health vulnerabilities facing HIV-affected caregivers and explore how the nature of such vulnerabilities informs our policy response. Both UNAIDS and UNICEF have provided initial guidance around HIV-sensitive social protection. Specifically, social policies and protections are defined as *HIV-sensitive* when they address vulnerabilities related to the epidemic and are inclusive of individuals who are at risk of or affected by HIV, without exclusively targeting these groups (UNAIDS, 2014; UNICEF, 2012; Yates et al., 2010). The term *HIV-specific* is reserved for those policies and protections which “focus exclusively on HIV and people living with and affected by HIV” (UNAIDS, 2014). Using these and other building blocks provided in UN guidance notes, we create a conceptual framework that is specific to HIV caregiving and can be used to more clearly link their needs to policy approaches.

**Figure 1.**
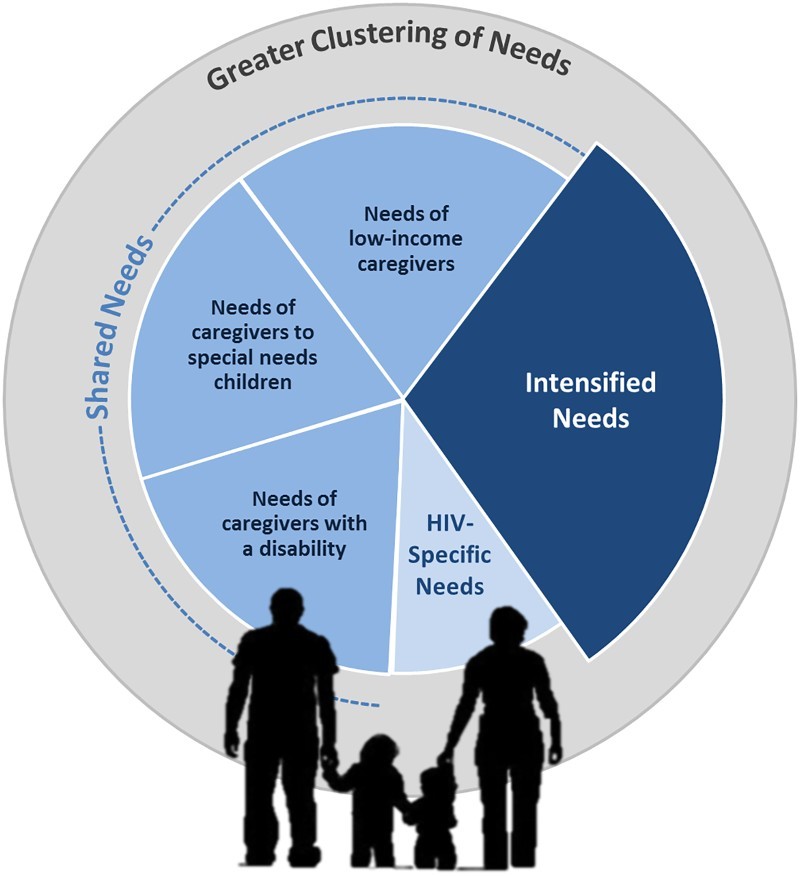
Conceptual framework to identify HIV-affected caregiver support needs and guide policy development.

*Shared needs*: UNICEF has argued that by focusing on the dimensions of vulnerability, rather than targeting specific groups, we can identify the most appropriate and equitable policy solutions (2012). Many dimensions of vulnerability for HIV-affected caregivers are widely shared with other caregiving groups: pervasive poverty; food insecurity; inability to cover school fees; ill health and/or disability; and limited access to safe childcare. Even orphan care is not specific to HIV: only a fraction of the 56 million orphans in sub-Saharan Africa are due to AIDS (UNICEF, 2013). In the conceptual framework (Figure 1), needs that cannot be distinguished from those of other caregiving populations are represented as shared needs.

In particular, the needs of HIV-affected caregivers closely resemble those of three other groups: caregivers living in poverty; caregivers supporting family members with special needs; and caregivers living with a disability. Like many in poverty, HIV-affected caregivers face an uphill battle to provide for their families. Many HIV-affected caregivers do not earn enough income to adequately feed and clothe their families (Heymann, Earle, Rajaraman, Miller, & Bogen, 2007; Kuo & Operario, 2010; Miller et al., 2006). They struggle to find safe, affordable childcare, and often lose income-generating work due to caregiving conflicts (Rajaraman, Earle, & Heymann, 2008). They need – but are rarely guaranteed – time off to tend to a sick child or consult with their teachers.

There are also striking commonalities between those caring for HIV-affected children and those caring for family members with physical, mental or intellectual disabilities. Children born with HIV often experience cognitive and developmental delays (Le Doaré, Bland, & Newell, 2012); those who are uninfected may none-the-less experience emotional and behavioral problems (Chi & Li, 2012). While HIV may play a causal role, the challenges facing these children and their caregivers mirror those of other populations. To address special needs, caregivers need additional financial resources and access to appropriate services, such as inclusive education and mental health counseling.

Finally, many HIV-affected caregivers are living with a disability or other health issue and need protections afforded to the millions of other caregivers who struggle with similar challenges. For some HIV-affected caregivers, disability is related to their own HIV status. The stigma surrounding disability interferes with caregivers’ ability to find and sustain employment; frequent or prolonged illness also jeopardizes earnings. Others develop health issues (e.g., depression) when they take on new caregiving roles (Ciesla & Roberts, 2001; Kuo & Operario, 2011). HIV-affected caregivers thus join a wider pool of individuals living with a disability or health issue, all of whom need equal rights in the workplace, paid leave to care for their own health, and access to specialized services.

From the above brief review, it is evident that many dimensions of vulnerability for HIV-affected caregivers are indistinguishable from those of other caregiving groups. In these instances, delivering social protection exclusively to HIV-affected families is counterproductive. Narrowly targeted services amplify stigma, are harder to deliver, often exclude large numbers in extreme need, and create competition for resources among civil society (UNAIDS, 2014). Thus, shared vulnerabilities are better addressed through universal programming. Such policies may be truly universal (e.g., paid parental leave); others may be available to caregivers and families that fall below a particular threshold of need (e.g., cash transfers).

*Unique needs*: It is also important to recognize where the needs of HIV-caregivers diverge. Below we discuss three unique features that are important in guiding HIV-sensitive policy: the extent to which vulnerability (1) is intensified among HIV-affected families, (2) clusters within HIV-affected families and (3) is specific to HIV-affected families.

First, HIV often exacerbates existing vulnerabilities. For example, poverty is intensified in HIV-affected families: medical bills drain resources and illness interferes with income-generating work. There may suddenly be many more dependent children to feed and clothe, further exacerbating economic and food insecurity (Kuo & Operario, 2010). For elderly caregivers, the HIV-related death of their own children means that they must not only raise grandchildren, but do so without the intergenerational transfers they might have otherwise received. These needs can still be met through universal programs, but require added sensitivity to the scale and nuance of HIV-related consequences. Elderly caregivers, for example, are not in the workforce and thus may require cash transfers to help them meet their HIV caregiving responsibilities. While transfers are relevant for the general population, such protections are of even greater importance in HIV-affected communities and should be prioritized in endemic contexts.

Second, HIV-affected families often face multiple, potentially compounding vulnerabilities. HIV-affected children commonly reside in the same household as HIV-infected family members, creating dual caregiving responsibilities (Kidman & Thurman, 2014). Far from being independent, vulnerabilities at the level of the household, caregiver and child are interwoven and compound one another. For instance, food scarcity amplifies caregiver depression (Littrell, Boris, Brown, Hill, & Macintyre, 2011); depression accelerates disease progression (Rabkin, 2008); and poor health among caregivers results in more poor health among children (Thielman, Ostermann, Whetten, Whetten, & O’Donnell, 2012). Thus, a sizable proportion of HIV-affected families will be overwhelmed and marginalized. These families need additional provisions to ensure access to universal protections. The help of a professional intermediary, such as a social worker, is likely needed to identify the most vulnerable families, to facilitate linkage to appropriate services, to coordinate between sectors, and to monitor progress. Focusing additional support on families with complex caregiving challenges thus ensures that social protections are inclusive of families affected by HIV, again without using HIV-targeting criteria.

Third and finally, there are vulnerabilities that are unique to HIV-affected caregivers, and which require specialized services to mitigate. Clearly, HIV-positive caregivers and children need timely access to quality medical care. Caregivers also need help caring for HIV-positive family members at home, support to disclose HIV status to children, and constitutional protections against HIV-related discrimination. The above needs are HIV-specific, and cannot be addressed through universal protections alone. We note that the list is short: we could identify very little vulnerability requiring HIV-specific responses. Moreover, there is ample grey area. For example, home-based care is critical to support HIV-infected patients, but such services are increasingly evolving to serve patients with a broad array of chronic or geriatric conditions (Aantjes, Quinlan, & Bunders, 2014). Thus, such services may still be integrated into primary health care systems, with adequate attention to ensure that such systems are capable of addressing HIV-specific needs (e.g., ART treatment regimens).

## The policy gap between what is needed and what is available

In this section, we highlight essential components of a social policy framework to support HIV-affected caregivers (see Table 1). Many of our recommendations coincide with those of intergovernmental agencies (UNAIDS, 2014; UNICEF, 2012). We build on the emerging discussion in two ways: first, we give specific examples of what policies are most critical to support caregiving to HIV-affected children based on the conceptual framework above; second, we provide the first systematic analysis of policies in heavily affected countries to identify critical gaps. We note that neither the summary of vulnerabilities above nor of policy solutions below is meant to be exhaustive; rather they illustrate a conceptual approach for meeting the needs of HIV-affected caregivers.

**Table 1.** Caregiver needs, supportive policies, and existing policy gaps in 25 highest HIV prevalence countries.

Target	Social policy	Policy gap	Notes
Economic security	Minimum wage	13 of 22 countries	In 12 countries the minimum wage is set too low to guarantee a worker and his/her dependent child would be above the $2 PPP/day global poverty line. In one country, there is no national minimum wage
	Cash family benefit	19 of 21 countries	In 12 countries, there are no known cash family benefits. In additional 7 countries, benefits are less than $20 PPP per month for a family with two preschool-age children
	Pensions for the elderly	16 of 21 countries	One country has no pension system. In an additional 15 countries, pensions are only provided through a contributory system, limiting income protection to workers in the informal economy and those who have had caregiving absences from work
	Financial support for families with disabled children	18 of 19 countries	Ten countries have no or limited family benefits. An additional eight countries provide family benefits, but do not have specific benefits for family with disabled children
	Short-term income protection during illness (leave available from 1st day)	3 of 23 countries	Three countries do not guarantee any income protection during illness (i.e., paid sick leave)
	Long-term income protection during illness (at least 26 weeks)	16 of 23 countries	In addition to those countries that do not guarantee any short-term sick leave, another 8 countries provide less than 26 weeks of paid leave
	Unemployment benefit	23 of 23 countries	None of the countries ensure unemployment benefits extend to workers in the informal economy. In 21 countries, unemployment is provided only through severance pay. The two countries that have unemployment benefits exclude self-employed workers
Work-family balance	Leave to care for sick children	22 of 25 countries	Sixteen countries do not have leave available specifically to meet children’s health needs. In four countries, leave for children’s health needs is limited to serious illnesses, hospitalizations, or imminent death. In one country leave is only available to mothers for children’s everyday health needs and in an additional country this leave is unpaid
	Leave to care for sick adults	23 of 25 countries	In 21 countries, there is no leave specifically to meet adult family members’ health needs. In additional two countries, leave is available but it is unpaid
	Leave to attend teacher conferences at school	17 of 24 countries	Seventeen countries have no form of leave that can be used to meet children’s educational needs (discretionary or family needs leave)
	ECD (age 0–3)	Not available	
Access to education	Free pre-primary school (age 4–5)	17 of 17 countries	None of the countries have free pre-primary education
	Free primary school	3 of 22 countries	Three countries report tuition in primary
	Free secondary school	14 of 20 countries	Eleven countries report charging tuition at the beginning of secondary and an additional three charge tuition before completion
	Inclusion of children with special needs	3 of 18 countries	One country has no public special education and an additional two countries only provide education for children with disabilities in separate schools
Access to health care	Constitutional guarantee to health	15 of 25 countries	Three countries have no constitutional provisions guaranteeing the right to health, medical services, or public health. In 12 countries, this provision is only aspirational
	Free mental health services for children and adults	Not available	
	Free HIV-related medical services (e.g., provision of ARVs; home-based care)	Not available	
Equal rights & discrimination	Constitutional guarantee of equal pay for women	20 of 25 countries	Only five countries constitutionally guarantee women equal pay for equal work. In an additional six, it is either aspirational or guaranteed broadly to citizens, but not specifically on the basis of gender
	Constitutional guarantee of protection from discrimination at work (general)	18 of 25 countries	Eighteen countries have no relevant constitutional provisions to protect all citizens or citizens with disabilities specifically from discrimination at work
	Constitutional protection of the right to education based on disability, health, or HIV status	18 of 25 countries	Five countries have no constitutional guarantee of the right to education. Fifteen countries guarantee citizens the right to education, but do not explicitly guarantee it to children with disabilities. One country aspires to guarantee the right to education for children with disabilities
	Constitutional protection from discrimination based on disability, health status, or HIV status	13 of 25 countries	In two countries, the constitution takes no approach to equality and non-discrimination based on disability or HIV status. In 13 countries, equality and non-discrimination is guaranteed to citizens but not specifically based on disability or HIV status. In an additional two countries, citizens with disabilities are guaranteed equal rights except for those that they are “unable, or not fully able, to enjoy or carry out”
Expanding social services	Family outreach and case management	Not available	
	Support for guardianship (e.g., birth registrations; streamlined administrative processes)	Not available	

Notes: The countries included are Angola, Botswana, Cameroon, Central African Republic, Chad, Congo, Cote d’Ivoire, Equatorial Guinea, Gabon, Guinea-Bissau, Kenya, Lesotho, Malawi, Mozambique, Namibia, Nigeria, Rwanda, South Africa, South Sudan, Swaziland, Togo, Uganda, United Republic of Tanzania, Zambia, and Zimbabwe. Missing policy data may occur for a variety of reasons, including if the full legislation is not available for a country or when it is not clear/contradictory. “Not available” indicates that globally comparative data on the policy were not available. For more information on the methods used to generate the policy data, please see http://worldpolicycenter.org/methodology.

We rank countries by adult HIV prevalence using 2014 UNAIDS estimates (2015). This results in the inclusion of the following countries: Angola, Botswana, Cameroon, Central African Republic, Chad, Congo, Cote d’Ivoire, Equatorial Guinea, Gabon, Guinea-Bissau, Kenya, Lesotho, Malawi, Mozambique, Namibia, Nigeria, Rwanda, South Africa, South Sudan, Swaziland, Togo, Uganda, United Republic of Tanzania, Zambia, and Zimbabwe. While our intention was not to focus on a particular region, the 25 countries with the highest prevalence are in fact all in Africa. Had we focused instead on the absolute number of adults living with HIV, our list of countries would have included others outside this region (e.g., Indonesia and Thailand).

For the top 25 countries ranked by prevalence, we analyze globally comparable policy data collected by the WORLD Policy Analysis Center between 2012 and 2014. Data were collected by reviewing a country’s constitution, legislation, policy, and social protection documents; secondary sources also included UN reports and country reports submitted to international bodies. Secondary sources were used when primary legislation was not available or when further clarification was required. Missing data result when legislation could not be obtained, when legislation was not clear or was contradictory, and/or when the country was not covered by key secondary sources. In Table 1, missing policy data are reflected in the denominator. Further detail on WORLD’s methodology is available at http://worldpolicycenter.org/methodology.

*Adequate time to provide care*: A core challenge is ensuring adequate time to provide care and to earn a living. We highlight several policies that would be instrumental in reshaping the workplace environment to be more conducive to caregiving. First, sick and family leave policies ensure that caregivers can attend to their children’s health needs without jeopardizing their livelihood. While these policies are relevant to all working caregivers, they are particularly salient in HIV-affected homes where either the child or another family member is HIV-positive. Leave policies are not common among the countries hardest hit by HIV: only three countries offer paid leave to care for sick children, and only two countries offer paid leave to care for sick adults. Caregivers also need time off from work to meet children’s educational needs, which again can be more time-consuming when the child has a disability. Seven countries offer some sort of leave that can be used for this purpose. Finally, caregivers with very young children need additional support to balance work and family responsibilities. Early childhood development (ECD) programs provide a safe, nurturing environment for children 0–3; they also provide essential coverage for working caregivers. For high-risk children especially, ECD programs play an important role in their healthy development and are fundamental for achieving equity in child outcomes (Daelmans et al., 2015). Globally comparable data is not currently available on publically provided 0–3 care.

*Economic security*: Addressing poverty for HIV-affected families, as for other families in poverty, begins with policies that raise earned income. We know that establishing an adequate minimum wage can help raise families out of poverty (Alaniz, Gindling, & Terrell, 2011; Gindling & Terrell, 2010). While directly benefiting those in the formal economy, raising the minimum wage also often raises the shadow minimum wage in the informal economy (e.g., Khamis, 2008; Kristensen & Cunningham, 2006). Almost all countries with a generalized epidemic have established a minimum wage. In more than half, however, a worker with one dependent child who earns the minimum wage will still fall below the global poverty line (see Table 1). Moreover, gender-equitable access to employment opportunities is critical for caregivers, who are disproportionately women (Govender, Penning, George, & Quinlan, 2011; Messer et al., 2010), yet only five countries constitutionally guarantee women equal pay for equal work.

Another critical concern is protecting income during an illness. Caregivers to HIV-affected children are more likely than other poor caregivers to be HIV-positive and to have health problems related to caregiving. Sick leave provides income stability during an illness, and ensures that there is a job to come back to when they recover. Of the high HIV prevalence countries with data, almost all guarantee leave from the first day of illness. There is room for further progress: just seven countries guarantee paid sick leave for a duration adequate to cover a serious illness. Finally, unemployment benefits can help ensure that hard-won gains are not quickly wiped away during an economic downturn. No country we reviewed offers comprehensive, government-sponsored unemployment benefits (see Table 1 for further detail). Twenty-one countries require that employers provide severance pay and two countries have government unemployment benefits; in both cases benefits exclude those in the informal economy.

The large numbers of HIV-affected children residing with elderly relatives suggests that pensions are also a critical mechanism for ensuring economic security. Most countries in our review had pensions, though these were predominately provided through a contributory system which excludes workers in the informal economy or who had long absences for caregiving. There is good evidence that cash transfers improve child nutrition, health and education in the short-term in poor families (including among HIV-affected families (Miller, Tsoka, Reichert, & Hussaini, 2010; Robertson et al., 2013; The Working Group on Social Protection for the Inter-Agency Task Team on Children and HIV and AIDS, 2007)); there is less robust evidence of their ability to lift families out of poverty in the long run. Public cash transfers have not been widely adopted in sub-Saharan Africa: only two countries guarantee an adequate cash benefit to poor families. Finally, families caring for special needs children may require additional financial resources; yet we could identify only one country that offered an explicit benefit for such families (Table 1).

*Access to Education*: Children who are orphaned or reside with HIV-positive parents are more likely to be out of school (Guo, Li, & Sherr, 2012). School fees are a major barrier, and caregivers need help covering this cost. Many community programs and some states offer HIV-affected children tuition bursaries, but universal programing can be more effective and equitable. Countries that have eliminated school fees have seen huge increases in both enrollment and inclusion (Deininger, 2003; Lucas & Mbiti, 2012). Most countries in our sample offer tuition-free primary school, but still require tuition for pre-primary (17 of 17 countries for which data is available) and for secondary school (14 of 20 countries). Finally, children born with HIV are more likely to have cognitive and developmental delays, placing them within a larger pool of children with special needs. The best educational outcomes are observed when special needs children are taught in the same classrooms as their peers (UNESCO, 2009). This is another area where substantial progress has been made: 15 hard hit countries already guarantee inclusive education within the public school system. While only seven countries have a constitutional protection from discrimination in education regardless of disability, this may partly reflect the year in which the constitution was penned.

*Access to Healthcare*: Caregivers need access to essential medical services and protection from the financial risks associated with illness. This is particularly true within HIV-affected families: caregiving – whether for orphaned children or the chronically ill – takes a physical and emotional toll (Bachman DeSilva et al., 2008; Ciesla & Roberts, 2001; Govender et al., 2011; Kuo & Operario, 2011; Littrell et al., 2011; Orner, 2006). HIV-affected children also experience more health challenges, ranging from tuberculosis to depression (Chi & Li, 2012; Cluver, Orkin, Moshabela, Kuo, & Boyes, 2013), and need timely access to appropriate health services. Universal health coverage (UHC) would go far to strengthen the caregiving environment. WHO member countries affirmed their commitment to UHC in 2005 (Carrin, Mathauer, Xu, & Evans, 2008). Globally comparable data on their progress towards this goal (e.g., public health insurance; free primary healthcare; free mental healthcare) is not available. Only 15 of the 25 countries reviewed have constitutional provisions guaranteeing the right to health (Table 1).

Almost by definition, a substantial proportion of caregivers and children in HIV-affected families will carry the virus. HIV-positive individuals require universal access to antiretroviral treatment and care, including home-based care. Adopting a family-centered model of HIV care (Richter, 2010), including co-locating pediatric and adult services, would benefit already overburdened caregivers. This is one of the rare instances in which HIV-specific services are necessary.

*Expanding Social Services*: As highlighted earlier, HIV-affected families have complex vulnerabilities, which may overwhelm their ability to access universal protections. Additional mechanisms are necessary to ensure that they are not left behind. Public social workers can identify the most vulnerable families, link them to appropriate services, and help them navigate complicated bureaucracies. Vulnerability among HIV-affected families is not static; vulnerabilities evolve as disease progresses and children age (e.g., the risk of sexual violence – especially for orphans – rises as girls mature (Kidman & Palermo, 2016)). Through regular contact with the family, social workers are well positioned to recognize and respond to emerging threats. While most countries already have public ministries charged with social welfare, they are often insufficient. For example, one study estimated that there were only 24 social workers per 100,000 people in South Africa (Earle-Malleson, 2009). Social workers must be adequately funded, capacitated, and made available on the scale required. Another hurdle in accessing public services is documentation, particularly among caregivers who are fostering children (Kuo & Operario, 2010). Compulsory birth and death registries, as well as streamlined procedures for establishing legal guardianship, would facilitate this process. Globally comparable data on social welfare investments or on compulsory registration systems are needed.

*The policy gap*: Table 2 presents an overview of the policy landscape for each country. More detailed data at the country level is available to policy-makers, civil society, and researchers at http://worldpolicycenter.org. In total, we explored 23 policies above, and have data available to analyze coverage for 19. No country has a comprehensive set of policies in place, either across or within substantive domains. Countries that stood out for making progress across many domains include South Africa and Kenya. Countries are most likely to have at least one policy for education (most offer tuition-free primary education) and for economic security (most guarantee short-term sick leave, though South Africa stands out for having a more robust package of economic provisions). While the most affected countries arguably have the greatest imperative to implement HIV-sensitive policies, they may face substantial obstacles, and they do not appear to have made greater policy progress.

**Table 2.** Existing policy landscape in the 25 highest prevalence countries, by country and domain.

Country	Adult (15–29) HIV prevalence	World Bank income level^a^	Known number of recommended social policies	At least one policy in each of the following domains				
				Economic	Work-family	Education	Health	Equal rights
Swaziland	27.7	Lower middle	4	✓		✓		
Botswana	25.2	Upper middle	5	✓		✓		
Lesotho	23.4	Lower middle	5	✓		✓		
South Africa	18.9	Upper middle	8	✓	✓	✓	✓	
Zimbabwe	16.7	Low	5	✓			✓	✓
Namibia	16	Upper middle	6	✓	✓	✓		
Zambia	12.4	Lower middle	3	✓		✓		
Mozambique	10.6	Low	4	✓		✓	✓	
Malawi	10	Low	6	✓		✓		✓
Uganda	7.3	Low	4	✓		✓		✓
Equatorial Guinea	6.2	High	4	✓				✓
Kenya	5.3	Lower middle	7	✓		✓	✓	✓
Tanzania, United Republic of	5.3	Low	6	✓	✓	✓		✓
Cameroon	4.8	Lower middle	5	✓	✓	✓		
Central African Republic	4.3	Low	5	✓	✓	✓		✓
Gabon	3.9	Upper middle	6	✓	✓	✓		
Guinea-Bissau	3.7	Low	0					
Cote d’Ivoire	3.5	Lower middle	4	✓	✓		✓	✓
Nigeria	3.2	Lower middle	4	✓		✓		
Rwanda	2.8	Low	7	✓		✓	✓	
Congo, Republic of	2.8	Lower middle	3	✓	✓	✓	✓	
South Sudan	2.7	Low	4			✓	✓	✓
Chad	2.5	Low	4	✓		✓		
Togo	2.4	Low	5	✓	✓	✓	✓	✓
Angola	2.4	Upper middle	7		✓	✓	✓	✓

^a^The World Bank divides economies into four categories (low, lower middle, upper middle, and high) using gross national income (GNI) per capita (The World Bank, 2016).

## Methodological limitations and knowledge gaps

We note several limitations to our approach. First, while the use of nationally comparative policy data is a real strength, there are several recommended policies for which no globally comparable data exist, including access to health, early childhood, and social services. Filling this information gap is a crucial first step towards prioritizing action. Second, the recommended policies rely on good governance, adequate resources, and well-functioning systems. We have data only on whether social policies to support HIV-affected families have been adopted; adequate data do not exist on how well they have been implemented. Moving forward, there should be sustained efforts to monitor not only policy development, but also implementation and ultimately impact. This is crucial to accelerating progress. It will also help policy-makers answer key questions, such as how to prioritize individual and groups of policies and actions. Third, universal policies may have a differential impact on HIV-affected caregivers. It is worth evaluating their success in achieving equitable coverage and benefit for HIV-affected caregivers (UNICEF, 2012).

## Opportunities for advancing policy

Both the UN and its member countries have committed to building strong families and to supporting childrearing (UN CRC article 181 (UN General Assembly, 1989)); this role is particularly salient in the wake of the HIV/AIDS epidemic. Caregivers to HIV-affected children need pathways to economic security, access to education and health care for their children, and workplace environments that support caregiving. This paper went beyond global commitments, and examined the national laws and policies in highly affected countries, focusing on those that matter most to HIV-related caregiving. We find there has been notable progress in some areas (e.g., short-term paid sick leave, free pre-primary education). However, a series of historical developments and economic constraints have impeded progress towards other internationally agreed upon targets.

Where social protections are missing, global assistance (financial, technical, and otherwise) and cross-country learning can help countries more quickly realize their commitments. For example, for countries still working toward tuition-free pre-primary and secondary education, external funding, from partners such as the Global Partnership for Education, may help until GDP grows sufficiently for internal resources to cover the cost. As a second example, global economic agreements can support country efforts to establish minimum wages above the global poverty line. In addition, cross-country learning is invaluable. Most countries already offer income protection when workers have short-term personal illnesses, demonstrating the feasibility of this approach and providing a platform for expansion, however many do not offer leave to care for children. Countries with similar economies have also been able to successfully implement paid leave to care for sick children or family members – a notable gap in highly affected countries – and could be valuable resources for countries interested in adopting similar legislation. Cross-sector learning and collaboration is equally critical. Just as HIV efforts have produced transferable lessons for other sectors (e.g., around chronic disease management), organizations working to advance the rights of other vulnerable caregiving groups (e.g., those living in poverty, with a disability, or supporting family members with special needs) could inform efforts to support HIV-caregivers. Building coalitions across sectors could not only facilitate information diffusion, but also give a more powerful voice to vulnerable caregivers.

Another notable area in which social protections are missing is in the informal economy. The policies described in this paper are concentrated in the formal economy, yet in many high prevalence countries, the majority of low-income workers are in the informal economy. This makes understanding the best coverage for the informal economy crucial. There are three primary ways in which the formal policies we describe can affect households with members laboring in the informal economy. First, households can have members both in the formal and informal economy. These households benefit even when policies only increase income and protection of some workers. Second, policies that target the formal economy can have positive consequences for the informal economy. Raising the minimum wage has been shown to lead to increases in the shadow minimum wage in the informal economy. For instance, in a study of 19 countries in Latin America and the Caribbean, 14 countries showed an increase in the informal wage after legally mandated increases in the minimum wage (Kristensen & Cunningham, 2006). There has not been a similarly extensive study in Africa, despite the need to understand whether the minimum wage could similarly boost informal wages in this region.

The third way in which the formal policies can affect those laboring in the informal economy is by being more inclusive. Policies – such as unemployment benefits, pensions, paid parental leave, and sick leave – can be designed in such a way as to explicitly include informal workers. For instance, unemployment benefits can be delivered through inclusive government insurance mechanisms, as opposed to employer-mandated severance pay. In our review, we find only two countries that offered government-sponsored unemployment benefits, and neither country extends such benefits to self-employed workers. Likewise, we find that three quarters of the countries with a government-sponsored pensions relied on contributory systems that exclude workers in the informal economy. Similar data on parental leave and sick leave policies are not available.

The voluntary and not-for-profit sectors make an invaluable contribution to innovation and service delivery – particularly for most marginalized groups. However, such organizations rarely have the resources to bring interventions to scale. Social protection has been championed as a strategy to realize HIV-objectives at a national scale, including mitigating the impact of the epidemic on children and families. To date, this has primarily been operationalized through HIV-targeted programming. National social policies offer a complementary strategy to provide meaningful protection to HIV-affected families. First, this approach embraces a more expansive view of social protection. Much of the current programming tries to mitigate the consequences of the epidemic by focusing on HIV-specific needs. At this stage in the response, additional strategies are needed to move from mitigation to empowerment. National protections can serve this purpose well, by building human capital and agency (Sabates-Wheeler & Devereux, 2008). Moreover, while local programs may share these aims, national policies are fundamentally the only ways to take such initiatives to scale.

Second, national policies can build resilience to future caregiving challenges. Rather than waiting for individuals to be profoundly affected by HIV and to experience negative consequences, strong protections can help create a reservoir of resilient caregivers. For instance, by creating sustainable livelihoods that can adapt to new caregiving roles, by ensuring that potential caregivers are healthy, and by reducing the financial burden of caregiving, national policies create a pool of caregivers able to support HIV-affected children from the onset.

Third and finally, there may be a synergistic relationship between HIV-specific programming and broad-based social protections. Targeted HIV programs may, because the core social protections are in place, have much greater impact than they would otherwise. For instance, most interventions (e.g., home visiting, ECD) aimed at caregivers require that they are both available and healthy enough to participate. As discussed above, national policies can facilitate both preconditions.

As the policy and knowledge gaps highlight, there are still many opportunities to advance national policies that matter for caregiving. As part of the HIV agenda, the global community can help national governments realize the potential of their commitments. If done well, this provides an opportunity to not only address the needs of children affected by HIV, but also to work collaboratively across sectors to improve the social conditions for all vulnerable families and children across the region.
